# Comparison of Retinal Layer Thickness and Capillary Vessel Density in the Patients with Spontaneously Resolved Acute Central Serous Chorioretinopathy

**DOI:** 10.3390/jcm10010045

**Published:** 2020-12-25

**Authors:** Kyu Jin Han, Hyeong Ju Kim, Je Moon Woo, Jung Kee Min

**Affiliations:** Department of Ophthalmology, Ulsan University Hospital, University of Ulsan College of Medicine, Ulsan 44033, Korea; hankj229@hanmail.net (K.J.H.); h0j8k29@naver.com (H.J.K.); limbus68@naver.com (J.M.W.)

**Keywords:** capillary vessel density, central serous chorioretinopathy, choriocapillaris, optical coherence tomography, optical coherence tomography angiography, vessel density

## Abstract

We investigate retinal layer thickness and capillary vessel density (VD) in the patients with central serous chorioretinopathy (CSC) who recovered spontaneously and evaluate the correlation between the changes in these values and visual outcomes using swept-source optical coherence tomography (SS-OCT) and OCT angiography (OCTA). This retrospective case–control study included 34 eyes of 34 patients with spontaneously resolved acute CSC. The changes in retinal layer thickness and capillary VD were examined using SS-OCT and OCTA after complete resolution of subretinal fluid (SRF). The fellow eyes and 34 healthy eyes were used as controls. In the eyes with CSC, the outer retinal layer was significantly thinner than in the eyes of fellow and healthy controls. The foveal avascular zone area and VDs in the superficial and deep capillary plexus in the eyes with CSC were not significantly different from those in the eyes of fellow and healthy controls. The VD of the choriocapillaris in the eyes with CSC was significantly lower than that in the eyes of fellow and healthy controls. Correlation analyses revealed that the outer retinal layer thickness and initial visual acuity were positively correlated with the final visual acuity. Furthermore, the initial SRF area and height were negatively correlated with the outer retinal layer thickness after SRF resolution. Attenuation of outer retinal layer thickness and decreased VD of the choriocapillaris were observed in the eyes with spontaneously resolved acute CSC. The outer retinal layer thickness could be an important visual predictor of CSC.

## 1. Introduction

Central serous chorioretinopathy (CSC) is a chorioretinal disease. Increased permeability of the choriocapillaris (CC), which in turn causes detachment of the neurosensory retina is caused due to dysfunction of the retinal pigment epithelium (RPE) [1,2]. The exact pathogenesis of CSC has not yet been fully elucidated; however, choroidal vascular alteration and RPE dysfunction have been implicated in the pathogenesis of CSC [3,4].

Optical coherence tomography (OCT) has made it possible to obtain high-resolution cross-sectional images of the retina in a noninvasive manner. The morphological changes that occur in acute CSC eyes comprise the formation of serous retinal detachment (SRD), RPE detachment, and RPE abnormalities. These changes have been clearly shown using OCT [1,5]. The outer retinal layers are relatively well-visualized and preserved [6]. Increased choroidal thickness associated with an increased choroidal vascular component in affected eyes is easily detectable with enhanced depth imaging OCT [7,8,9].

OCT angiography (OCTA) is a relatively new dye-free depth-oriented angiographic imaging technique with which vascular changes in CSC can be objectively and qualitatively analyzed [10]. The three-dimensional (3D) vascular map obtained can be automatically segmented to visualize and assess the retinal and choroidal vessels at specific depths [11]. Because of its inability to detect blood flow, OCTA is considered unsuitable for accurately detecting leakage points in CSC. However, OCTA can detect choroidal neovascularization (CNV) in CSC even in the absence of exudative activity [12].

Intervention is required in the case of chronic or recurrent CSC because the recurrent and chronic nature of this condition can result in an irreversible and severe visual loss secondary to persistent subretinal fluid (SRF) as well as atrophy of the RPE and photoreceptors [13,14]. However, most of the acute CSC cases resolve spontaneously in 2–3 months, with visual acuity returning close to premorbid levels [15]. Thus, there are insufficient data to clearly explain the relationship between visual outcomes and chorioretinal changes in the patients with spontaneously resolved acute CSC.

Therefore, we evaluated the relationship between retinal and choroidal changes, including retinal layer thickness, foveal avascular zone (FAZ) area, and capillary vessel density (VD), and visual outcomes in the patients with spontaneously resolved CSC. Additionally, the relationship between the SRF height and area and retinal and choroidal changes in acute CSC patients was evaluated using swept-source OCT (SS-OCT) and OCTA.

## 2. Materials and Methods

### 2.1. Study Participants

**Figure 1:**
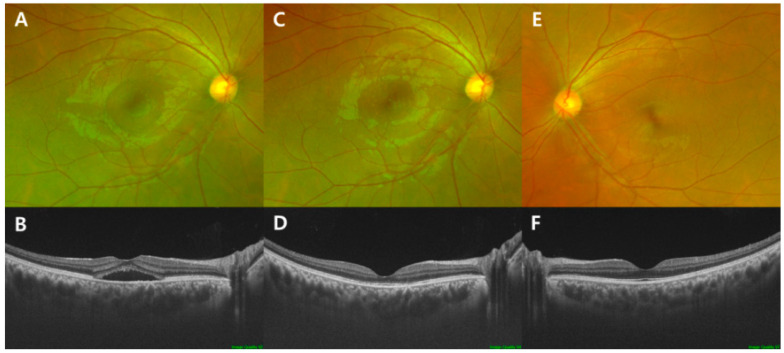
Fundus photography and optical coherence tomography (OCT) images in a 34-year-old man with acute central serous chorioretinopathy (CSC) showing serous retinal detachment (SRD) at the initial examination ((**A**) fundus photography; (**B**) OCT image), spontaneous resolution of subretinal fluid (SRF) after 1 month ((**C**) fundus photography; (**D**) OCT image)), and unaffected fellow eye ((**E**) fundus photography; (**F**) OCT image). The mean logarithm of the minimal angle of resolution (logMAR) best-corrected visual acuity was 0.00 (Snellen equivalent: 20/20) at the initial SRD phase and 0.00 (20/20) after spontaneous SRF resolution. Visual acuity in the fellow eye was 0.00 (20/20).

The medical records of the patients (34 eyes of 34 patients) who had been diagnosed with treatment-naïve CSC between 1 December 2016, and 31 October 2019, at the Department of Ophthalmology of Ulsan University Hospital, were reviewed. All patients underwent a baseline ophthalmic examination that included best-corrected visual acuity (BCVA) measurement, slit-lamp examination, fundus examination, SS-OCT, and OCTA. CSC was diagnosed based on the detection of SRF in the macula using SS-OCT (Figure 1). OCTA confirmed that CNV and branching vascular networks were not present in these patients.

Only patients with SRD duration of <6 months and complete resolution of SRF were included in the analyses. Patients with a known history of CSC or any treatment for CSC such as intravitreal anti-vascular endothelial growth factor injection and/or focal laser treatment and/or photodynamic therapy were excluded. Patients were also excluded if they had any preexisting ocular disease (e.g., glaucoma, uveitis, macular degeneration, retinal vascular disease, and severe media opacities) known to affect visual acuity or high myopia (near-sightedness of −6.00 diopters or more). In addition, patients with previous ocular surgery, including cataract surgery, or previous retinal surgery, such as surgery for the epiretinal membrane, macular hole, and rhegmatogenous retinal detachment (RRD), were excluded. All patient examinations were performed at monthly intervals until complete disappearance of SRF. To minimize the segmentation and measurement errors of OCT and OCTA, the patients with subRPE fluid at 3 mm diameter inner circle in nine Early Treatment Diabetic Retinopathy Study (ETDRS) subfields at the initial visit were excluded.

Measurements obtained from the unaffected fellow eyes were also analyzed. The control group (34 eyes of 34 subjects) was selected as healthy subjects who visited to the ophthalmology outpatient clinic. They had a similar gender and age composition to the study group, and they did not have any ocular disease.

### 2.2. Optical Coherence Tomography and Angiography Imaging Protocols

We used an SS-OCT and OCTA system (DRI OCT-1, Atlantis; Topcon Corporation, Tokyo, Japan). The SS-OCT and OCTA images were analyzed using the automated segmentation software embedded in the OCT instrument (IMAGEnet 6 v1.22, Topcon, Tokyo, Japan).

The wide 3D scans produced maps that consisted of concentric circles with diameters of 1, 3, and 6 mm and provided automated thickness analyses that included the automated segmentation of each retina into nine ETDRS subfields. Total retinal layer thickness was defined as the distance from the internal limiting membrane (ILM) to the RPE outer segment, and ganglion cell layer (GCL) ++ thickness was defined as the distance from the ILM to the inner plexiform layer (IPL). We defined outer retinal layer thickness as the difference between total retinal layer thickness and GCL ++ thickness and used average values in the inner circle with a diameter of 3 mm.

**Figure 2:**
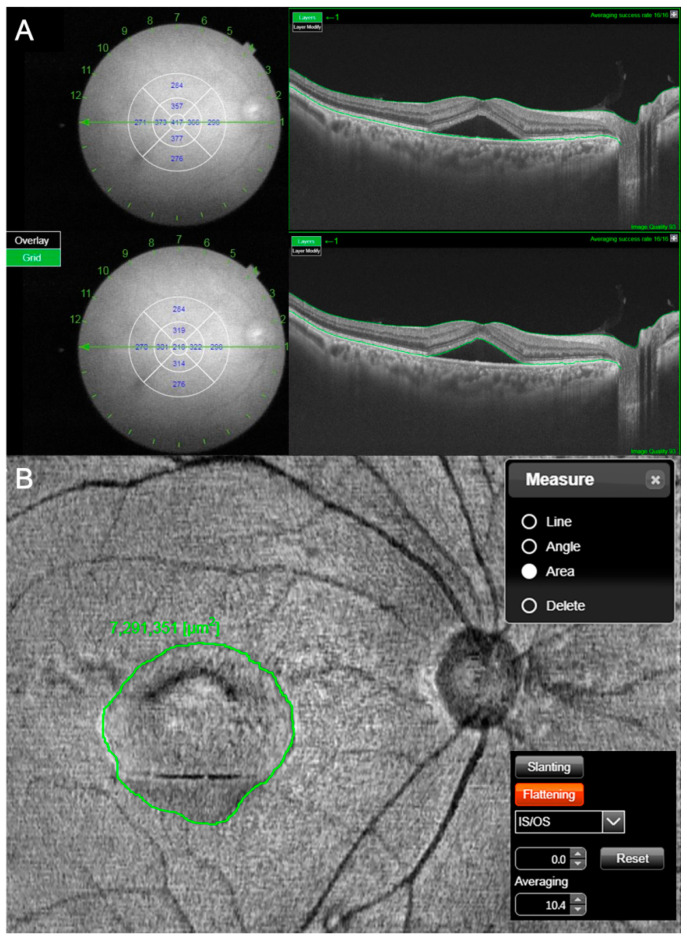
Measurements of subretinal fluid (SRF) height and area were calculated at the initial visit using the 12 radial wide scan and three-dimensional (3D) wide continuous swept-source optical coherence tomography (SS-OCT) mode. The SRF height was defined as the difference between mean total retinal thickness (**A**, top image) and mean neurosensory retinal thickness (**A**, bottom image) at 1 mm diameter center circle in nine Early Treatment Diabetic Retinopathy Study subfields. The measurements of each layer thickness were performed by semi-automated segmentation with manual corrections using the 12 radial wide scan (**A**). For measurement of SRF area, the 3D OCT scans were reconstructed as en face images. The reference slabs were aligned and flattened to the level of the inner/outer segment. The SRF area was defined as the area inside with contrast change at this level (**B**).

SRF height and area were measured at the initial visit using the 12 radial wide scan and 3D wide continuous SS-OCT mode. The SRF height was defined as the difference between mean total retinal thickness (Figure 2A, top image) and mean neurosensory retinal thickness (Figure 2A, bottom image) at 1 mm diameter center circle in nine ETDRS subfields. The measurements of each layer thickness were performed by semi-automated segmentation with manual corrections using the 12 radial wide scan. To measure SRF area, the 3D OCT scans were reconstructed as *en face* images using embedded image software. The reference slabs were aligned and flattened to the level of inner / outer segment at photoreceptor layer. SRF area was defined as the area inside with contrast change in this level (Figure 2B).

**Figure 3:**
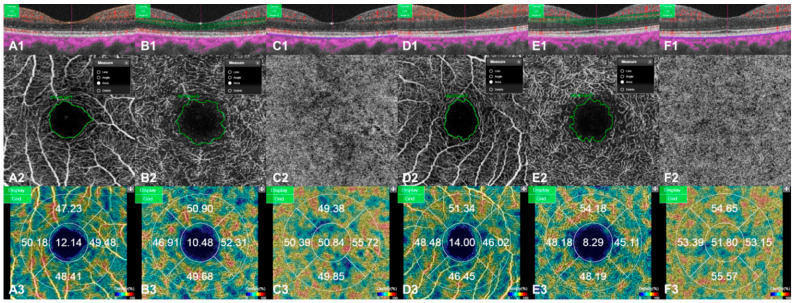
Measurements of the foveal avascular zone (FAZ) area and capillary vessel density (VD) were conducted using swept-source optical coherence tomography angiography (SS-OCTA). En face images were generated for retinal vascular networks: superficial capillary plexus (SCP), deep capillary plexus (DCP), and choriocapillaris (CC), after spontaneous resolution of subretinal fluid (SRF) 1 month later (**A1**–**C3**), and in unaffected fellow eyes (**D1**–**F3**). The cross-sectional scans show the corresponding segmentation layers and intact inner or outer segment junction (**A1**–**F1**). The FAZ area was defined as the area inside the central border of the capillary network and determined by manually outlining the inner border of foveal capillaries using the OCTA system software (**A2**,**B2**,**D2**,**E2**). The VD maps consisted of concentric circles with diameters of 1 and 3 mm. Foveal and parafoveal VDs of SCP, DCP, and CC were measured automatically (**A3**–**F3**).

FAZ area and capillary VD were measured using an SS-OCTA system. All OCTA scans were conducted by acquiring a 3 × 3 mm macular size. En face images were generated for retinal vascular networks: superficial capillary plexus (SCP), deep capillary plexus (DCP), and CC. SCP comprised capillaries from 2.6 µm below ILM to 15.6 µm below IPL. DCP extends from 15.6 to 70.2 µm below IPL. CC comprises 10.4 µm-thick capillaries to the RPE–Bruch’s membrane. The area inside the central border of the capillary network was considered as the FAZ area and was measured by manually outlining the inner border of foveal capillaries using the OCTA system software. The FAZ area was automatically calculated, and the average of the two measurements was used for analyses [16]. The VD was defined as the percentage area occupied by the microvasculature of each retinal vascular network at the center of the fovea. The software automatically produced maps consisting of concentric circles with diameters of 1 and 3 mm. Foveal and parafoveal VDs of the SCP, DCP, and CC were measured automatically (Figure 3) [17].

Measurements were obtained by the same trained examiner, and all OCT and OCTA scans were reviewed by a retinal specialist (J.K.M.) to ensure that they had sufficient quality and adequately visualized the retinal layers. The SRF height and area and FAZ area in the SCP and DCP were independently graded by two investigators (K.J.H. and J.K.M.). Because OCT and OCTA can involve some artifacts, and segmentation and measurement errors may occur in the presence of SRF, we only measured retinal layer thicknesses and VDs in the eyes with complete SRF resolution without subRPE fluid in the inner circle with a diameter of 3 mm [18].

### 2.3. Statistical Analyses

Continuous variables were expressed as the mean ± standard deviation. All BCVA values were converted to logarithm of the minimal angle of resolution (logMAR) values before performing the data analyses. Kruskal Wallis test and Mann Whitney U-test were used to compare differences in the mean values among groups (CSC eyes versus Fellow eyes versus healthy eyes). Spearman’s correlation analyses were performed to analyze the relationship among variables. All statistical analyses were performed using SPSS statistical software (SPSS Statistics 21.0, IBM, Chicago, IL, USA). A *p*-value of < 0.05 indicated a statistically significant difference.

## 3. Results

### 3.1. Baseline Patient Demographic Parameters and Clinical Characteristics

The 34 patients (24 men and 10 women) with a mean age of 47.47 ± 8.40 years were included in this study. The mean BCVA (logMAR) increased from 0.23 ± 0.23 at baseline to 0.03 ± 0.06 (*p* < 0.001) after complete resolution of SRF. The mean duration of SRD was 3.15 ± 1.33 months. The SRF area was 11.63 ± 8.30 mm^2^ and the SRF height was 161.24 ± 115.57 µm at the initial examination. The healthy subjects (24 men and 10 women) with a mean age of 48.32 ± 5.98 years were included as control (*p* = 0.403) (Table 1).

### 3.2. Comparison of Ocular Characteristics

The eyes with CSC showed statistically lower BCVA than both the fellow and healthy control eyes, although the visual acuity was improved significantly more than the initial visual acuity after complete SRF resolution (*p* = 0.002). Incomplete visual recovery (<0.00 logMAR) was observed in 8 of the 34 eyes (23.5%) with CSC. Both total and outer retinal layer thicknesses were significantly decreased in the eyes with CSC than in the fellow (*p* < 0.001 and *p* < 0.001, respectively) and healthy control eyes (*p* = 0.004 and *p* < 0.001, respectively). GCL ++ thickness in the CSC eyes was not significantly different from that in the fellow and healthy control eyes. The FAZ areas in both the SCP and DCP in eyes with CSC were not significantly different from those in the fellow and healthy control eyes. With regard to VDs of each capillary plexus, only the VDs of the CC (both center, 1 mm, and whole, 3 mm) in the CSC eyes were significantly lower than those in the fellow (*p* < 0.001 and *p* = 0.003, respectively) and control eyes (*p* = 0.002 and *p* = 0.022, respectively). The VDs of the SCP and DCP (both center, 1 mm, and whole, 3 mm) in the eyes with CSC were not significantly different from those in the fellow and control eyes. In comparison between the fellow eyes and the healthy control eyes, there was no statistically significant difference except for the outer retinal layer thickness. The outer retinal layer thickness in the fellow eyes was measured to be thicker than the healthy control eyes (*p* = 0.023) (Table 2).

**Figure 4:**
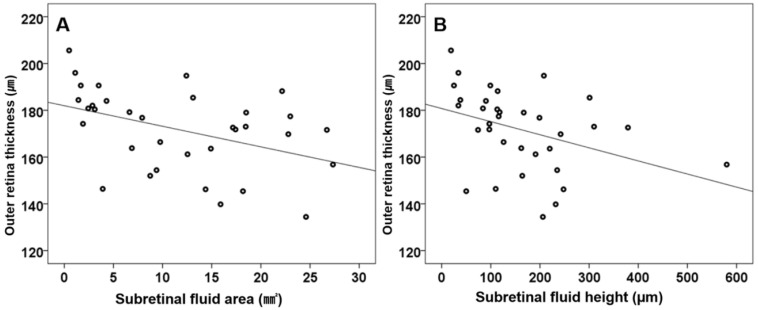
Relationship between the amount of subretinal fluid (SRF) (area and height) and outer retinal layer thickness, as determined using Spearman’s correlation coefficient analyses, in the eyes with acute central serous chorioretinopathy (CSC), showing serous retinal detachment (SRD) at the initial examination. The SRF area was moderately and negatively correlated with outer retinal layer thickness (*ρ* = −0.510, *p* = 0.002; (**A**)). The SRF height was also moderately and negatively correlated with outer retinal layer thickness (*ρ* = −0.472, *p* = 0.005; (**B**)).

Regarding the correlations between baseline clinical characteristics and final BCVA, better initial BCVA showed a significant positive correlation with better final BCVA (*ρ* = 0.436, *p* = 0.010). Correlation analyses also revealed that outer retinal layer thickness was positively correlated with final BCVA (logMAR) in the eyes with CSC (*ρ* = −0.513, *p* = 0.002) (Table 3). In addition, the SRF area and height at baseline were negatively correlated with outer retinal layer thickness (*ρ* = −0.510, *p* = 0.002 and *ρ* = −0.472, *p* = 0.005, respectively) (Figure 4). The interobserver repeatability for CSC eyes, fellow and control data were good for DCP FAZ area measurements (ICC = 0.857, 0.899 and 0.888 respectively) and excellent for SRF area & height and SCP FAZ area measurements (ICC = 0.929, 0.922, 0.921, 0.935, and 0.926, respectively; Table 4).

## 4. Discussion

The CSC is often self-limiting, and spontaneous resolution and complete fluid reabsorption often occur. The visual prognosis is good in 90%–95% of cases, and visual acuity returns to normal within a few months following SRF resolution [19]. Thus, patient observation and reassurance are considered the best treatment course. With the advent of technology, the valuable quantitative information from OCT and OCTA systems could greatly enhance our understanding of outer retinal diseases and would allow clinicians to confirm their improvement or progression [20,21].

In this study, we investigated retinal layer thickness and capillary VD in the patients with acute CSC after spontaneous recovery. FAZ areas and VDs of both the SCP and DCP were not significantly different from those in the fellow and healthy control eyes and had no correlation with BCVA in the eyes with CSC after complete SRF resolution. In a previous study, Mao et al. reported no significant differences in VD (of both SCP and DCP) and FAZ area between eyes with acute CSC and control eyes, which is consistent with our results. They also reported that chronic CSC was associated with decreased VD and expanded FAZ area and that both were correlated with poor visual acuity [22]. Typically, in healthy individuals, the FAZ area is not associated with visual acuity, whereas in some disease states such as diabetic retinopathy, retinal vein occlusion, and RRD, a larger FAZ area may be correlated with lower visual acuity [21,23].

Unlike VDs of the SCP and DCP, only VD of the CC showed a significant difference between the eyes with CSC and the eyes of fellow and healthy controls. Moreover, the outer retinal layer was thinner in the eyes with CSC than in the eyes of fellow and healthy controls, and GCL ++ thickness was not significantly different between the eyes with CSC and eyes of fellow and healthy controls. These results showed that the difference in total retinal layer thickness was because of the attenuation of outer retinal layer thickness, including outer plexiform layer and outer nuclear layer (ONL). Despite the fact that fellow eyes may not always be completely normal, characteristic findings of CSC, such as abnormal permeability of the vessels in the choroid, are often observed in both eyes, and the outer retinal layer thickness in the eyes with CSC was significantly decreased compared with that in the fellow eyes.

In this study, the SRF area and height were negatively correlated with outer retinal layer thickness. We believe that attenuation of outer retinal layer thickness indicates photoreceptor cell loss and amount of SRF influences photoreceptor damage. SRF contains inflammatory cytokines that trigger and enhance inflammatory and immune responses [24]. SRF also physically separates photoreceptor cells from the RPE, which disrupts the mutual metabolism. Photoreceptor cell loss in CSC may be attributed to altered renewal of the outer segment and increases in oxidative stress, which may lead to apoptosis of photoreceptor cells. The apoptosis of photoreceptor cells could initiate in 1–3 days and progress until SRF resolution or continue after resolution [25]. Wang et al. demonstrated that foveal attenuation in the reattached retina is associated with the duration of symptoms, indicating that the microstructural changes resulting from foveal detachment progress more when the detachment is more prolonged [26]. Several studies reported that the ONL thickness was negatively correlated with symptom duration and that the ONL thickness increased after treatment. These previous studies also reported that ONL thickness was positively correlated with BCVA [27,28,29,30]. However, in the present study, we were unable to show any correlation between symptom duration and outer retinal layer thickness. This suggests that the amount of SRF rather than the duration of SRF plays an important role in outer retinal damage in the acute phase (<6 months duration). Cennamo et al. and Qu et al. reported that VD of the CC was much lower in the affected eyes with SRF than in the fellow eyes [31,32]. In another study, Cennamo et al. reported that the responder group showed a significant increase in VD of the CC after photodynamic therapy [17]. These findings suggest that VD of the CC decreases in the presence of SRF and partially recovers after SRF resolution. However, we were unable to find a significant relationship between VD of the CC and BCVA, unlike a previous study on chronic CSC [22]. Collectively, we can imply that the outer retina may be damaged by inflammatory cytokines and impaired metabolism and is more vulnerable than the inner retina in acute CSC patients. Although the precise relationship between decreased VD of the CC and outer retinal layer thickness could not be determined in this study, decreased choriocapillary circulation may influence the permanent impairment or delayed restoration of the outer retina after SRF resolution. Therefore, more damaged choriocapillary circulation in the eyes with chronic CSC might show significant relationships between VD of the CC and outer retinal layer thickness or BCVA [33].

Our study has several limitations. First, this study has a retrospective design, and we cannot exclude recall bias. Second, this study has a relatively small sample size; thus, we need to be careful in interpreting statistical significance. Unlike previous studies, we performed not only the comparison with healthy control eyes, but also the comparison analysis with the fellow eyes, thereby minimizing bias due to other factors.

## 5. Conclusions

The results suggest that outer retinal layer thickness is predictive of visual outcomes following complete resolution of SRF in the eyes with acute CSC. While inner retinal layer thickness and VDs of the SCP and DCP are generally preserved, outer retinal layer thickness and VD of the CC are decreased after spontaneous SRF resolution in the eyes with acute CSC. Therefore, we suggest that choroidal vascular alteration and RPE dysfunction cause increased SRF amount and decreased VD of the CC, both of which lead to outer retinal ischemia. Ischemic damage to the outer retina in the fovea may result in incomplete visual recovery following complete SRF resolution in the patients with CSC, even in a short-term period of <6 months.

## Figures and Tables

**Table 1:** Demographic and clinical characteristics of patients at baseline.

Characteristic	Eyes with CSC (*n* = 34)
Age (years)	47.47 ± 8.40
Male/female	24/10
Symptom duration (months)	3.15 ± 1.33
Spherical equivalent (diopter)	−0.67 ± 1.99
Initial BCVA (logMAR)	0.23 ± 0.23
SRF area (mm^2^)	11.63 ± 8.30
SRF height (µm)	161.24 ± 115.57

BCVA: best-corrected visual acuity; CSC: central serous chorioretinopathy; logMAR: logarithm of the minimal angle of resolution.

**Table 2:** Comparison of retinal layer thickness, FAZ area, and vessel density between the eyes with CSC, fellow and healthy control eyes.

	Eyes with CSC (*n* = 34)	Fellow Eyes (*n* = 34)	Healthy Control Eyes (*n* = 34)	*p* *	*p* ^†^	*p* ^‡^	*p* ^§^
Final BCVA (logMAR)	0.03 ± 0.06	0.00 ± 0.00	0.00 ± 0.01	0.002	0.001	0.035	0.079
Spherical equivalent (diopter)	−0.67 ± 1.99	−0.82 ± 2.07	−0.74 ± 1.62	0.758	0.618	0.777	0.479
Total retinal layer thickness (µm)	275.81 ± 19.92	296.15 ± 13.52	290.15 ± 15.37	<0.001	<0.001	0.004	0.095
GCL++ thickness (µm)	104.08 ± 18.72	103.68 ± 9.09	102.77 ± 8.70	0.883	0.951	0.681	0.654
Outer retinal layer thickness (µm)	171.72 ± 17.21	192.47 ± 7.58	187.38 ± 10.84	<0.001	<0.001	<0.001	0.023
SCP FAZ area (mm^2^)	0.409 ± 0.111	0.394 ± 0.118	0.388 ± 0.146	0.742	0.715	0.432	0.715
DCP FAZ area (mm^2^)	0.721 ± 0.148	0.663 ± 0.158	0.653 ± 0.148	0.120	0.101	0.058	0.974
SCP vessel density center (%)	16.46 ± 4.02	17.28 ± 3.87	17.97 ± 4.96	0.356	0.419	0.171	0.461
SCP vessel density whole (%)	41.98 ± 1.48	42.05 ± 1.59	42.33 ± 1.78	0.896	0.888	0.641	0.768
DCP vessel density center (%)	14.31 ± 3.60	13.82 ± 3.94	14.68 ± 5.10	0.904	0.715	0.927	0.686
DCP vessel density whole (%)	42.52 ± 2.13	42.54 ± 2.13	42.92 ± 2.04	0.700	0.837	0.404	0.572
CC vessel density center (%)	47.77 ± 4.67	51.54 ± 3.27	51.29 ± 3.35	<0.001	<0.001	0.002	0.672
CC vessel density whole (%)	52.14 ± 1.46	52.89 ± 0.77	52.79 ± 1.23	0.009	0.003	0.022	0.934

Data are presented as mean ± standard deviation. *p*-values are derived using the Kruskal Wallis test (* indicates eyes with CSC vs. fellow eyes vs. healthy control eyes). *p*-values are derived using the Mann–Whiney U test (^†^ eyes with CSC vs. fellow eyes, ^‡^ eye with CSC vs. healthy control eyes, ^§^ fellow eyes vs. healthy control eyes). BCVA: best-corrected visual acuity; GCL: ganglion cell layer; FAZ: foveal avascular zone; SCP, superficial capillary plexus; DCP: deep capillary plexus; CC: choriocapillaris; CSC: central serous chorioretinopathy; logMAR: logarithm of the minimal angle of resolution.

**Table 3:** Correlations between OCT and OCTA parameters and BCVA in the patients with CSC.

	Final BCVA *ρ* (*p*-Value)	Initial BCVA *ρ* (*p*-Value)	Outer Retina Thickness *ρ* (*p*-Value)	SCP FAZ Area *ρ* (*p*-Value)	DCP FAZ Area *ρ* (*p*-Value)	SCP VD Center *ρ* (*p*-Value)	DCP VD Center *ρ* (*p*-Value)	CC VD Center *ρ* (*p*-Value)
Final BCVA *ρ* (*p*-value)	1							
Initial BCVA *ρ* (*p*-value)	0.436 (0.010)	1						
Outer retinal layer thickness *ρ* (*p*-value)	−0.513 (0.002)	−0.505 (0.002)	1					
SCP FAZ area *ρ* (*p*-value)	−0.33 (0.855)	0.074 (0.679)	−0.292 (0.094)	1				
DCP FAZ area *ρ* (*p*-value)	0.078 (0.661)	0.150 (0.396)	−0.191 (0.278)	0.034 (0.080)	1			
SCP VD center *ρ* (*p*-value)	0.051 (0.774)	−0.172 (0.331)	0.320 (0.065)	−0.913 (<0.001)	−0.391 (0.022)	1		
DCP VD center *ρ* (*p*-value)	0.127 (0.474)	0.066 (0.710)	−0.028 (0.877)	−0.820 (<0.001)	−0.442 (0.009)	0.739 (<0.001)	1	
CC VD center *ρ* (*p*-value)	−0.091 (0.610)	−0.238 (0.175)	0.013 (0.463)	−0.231 (0.188)	−0.327 (0.059)	0.321 (0.064)	0.213 (0.226)	1

*ρ* and *p*-values are derived from Spearman’s rank test. BCVA: best-corrected visual acuity; FAZ: foveal avascular zone; SCP: superficial capillary plexus; DCP: deep capillary plexus; CC: choriocapillaris; VD: vessel density; OCT: optical coherence tomography; OCTA: OCT angiography; CSC: central serous chorioretinopathy.

**Table 4:** Repeatability of SRF area and height, superficial and deep foveal avascular zone area measurements.

	SRF Area	SRF Height	SCP FAZ Area with CSC Eyes	DCP FAZ Area with CSC Eyes	SCP FAZ Area with Fellow Eyes	DCP FAZ Area with Fellow Eyes	SCP FAZ Area with Control Eyes	DCP FAZ Area with Control Eyes
ICC	0.929	0.922	0.921	0.857	0.935	0.899	0.926	0.888
*p*-value	<0.001	<0.001	<0.001	<0.001	<0.001	<0.001	<0.001	<0.001

Measurements were made by two independent examiners using built-in optical coherence tomography and angiography system software (IMAGEnet 6). DCP: deep capillary plexus; FAZ: foveal avascular zone; ICC: intraclass correlation coefficient; SCP: superficial capillary plexus; SRF: subretinal fluid; CSC: central serous chorioretinopathy.

## Data Availability

The data generated during and/or analyzed the current study are available from the corresponding author on reasonable request.
